# BMC Caller: a webtool to identify and analyze bacterial microcompartment types in sequence data

**DOI:** 10.1186/s13062-022-00323-z

**Published:** 2022-04-28

**Authors:** Markus Sutter, Cheryl A. Kerfeld

**Affiliations:** 1 Environmental Genomics and Systems Biology and Molecular Biophysics and Integrated Bioimaging Divisions, Lawrence Berkeley National Laboratory, Berkeley, CA 94720 USA; 2 MSU-DOE Plant Research Laboratory, Michigan State University, East Lansing, MI 48824 USA; 3 Department of Biochemistry and Molecular Biology, Michigan State University, East Lansing, MI 48824 USA

**Keywords:** Bacterial microcompartment, Protein HMM profile, Protein sequence analysis, Metabolosome, Carboxysome

## Abstract

Bacterial microcompartments (BMCs) are protein-based organelles found across the bacterial tree of life. They consist of a shell, made of proteins that oligomerize into hexagonally and pentagonally shaped building blocks, that surrounds enzymes constituting a segment of a metabolic pathway. The proteins of the shell are unique to BMCs. They also provide selective permeability; this selectivity is dictated by the requirements of their cargo enzymes. We have recently surveyed the wealth of different BMC types and their occurrence in all available genome sequence data by analyzing and categorizing their components found in chromosomal loci using HMM (Hidden Markov Model) protein profiles. To make this a “do-it yourself” analysis for the public we have devised a webserver, BMC Caller (https://bmc-caller.prl.msu.edu), that compares user input sequences to our HMM profiles, creates a BMC locus visualization, and defines the functional type of BMC, if known. Shell proteins in the input sequence data are also classified according to our function-agnostic naming system and there are links to similar proteins in our database as well as an external link to a structure prediction website to easily generate structural models of the shell proteins, which facilitates understanding permeability properties of the shell. Additionally, the BMC Caller website contains a wealth of information on previously analyzed BMC loci with links to detailed data for each BMC protein and phylogenetic information on the BMC shell proteins. Our tools greatly facilitate BMC type identification to provide the user information about the associated organism’s metabolism and enable discovery of new BMC types by providing a reference database of all currently known examples.

**Supplementary Information:**

The online version contains supplementary material available at 10.1186/s13062-022-00323-z.

## Background

**Fig. 1:**
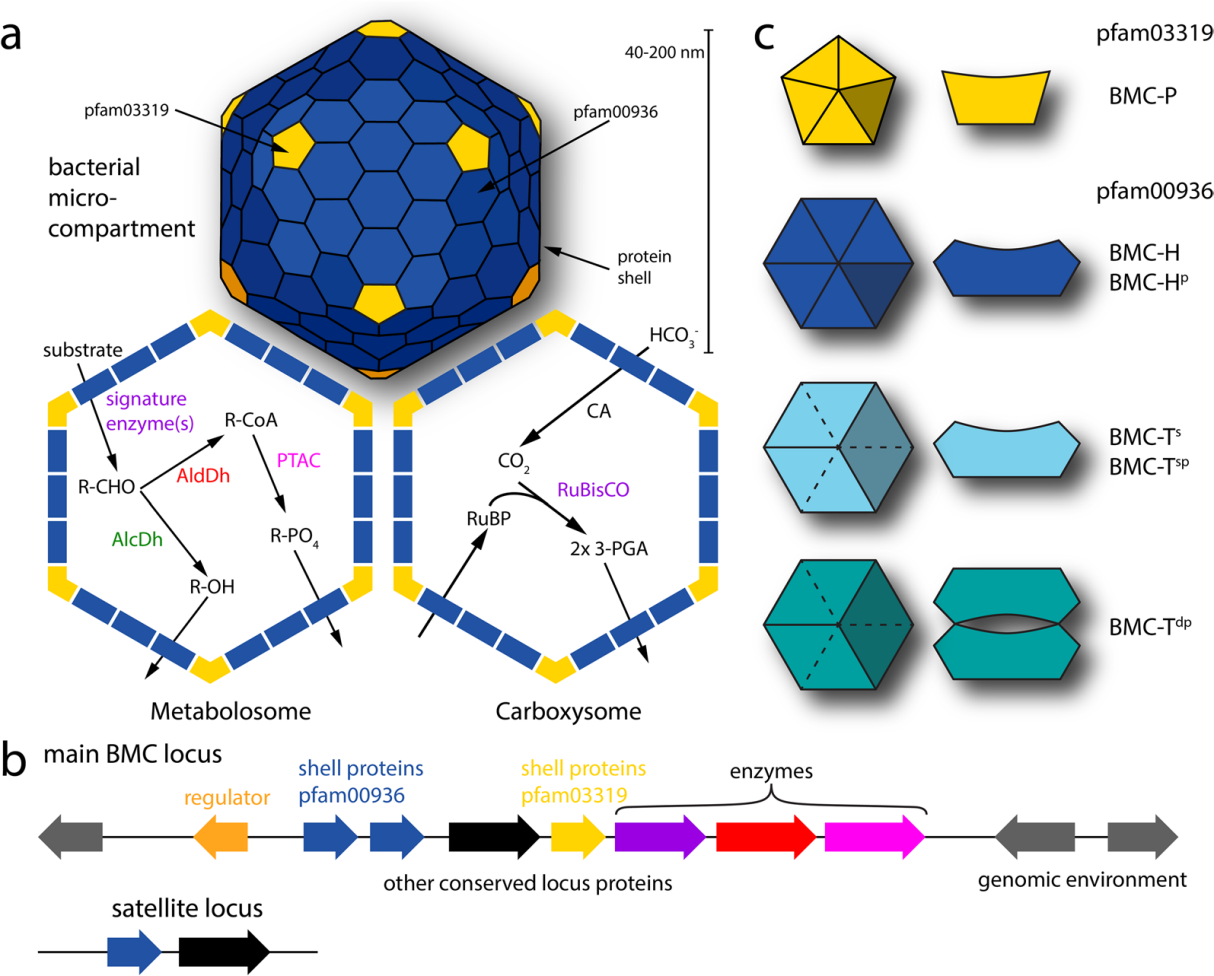
Overview of bacterial microcompartment structure and function. **a** General microcompartment properties and details of metabolosome and carboxysome reaction pathways. **b** Generalized BMC locus organization. **c** Types of BMC shell proteins. Color scheme used here and in BMC Caller analysis for genes/proteins: yellow: BMC-P, blue: BMC-H/ BMC-H^p^, light blue: BMC-T^s^/BMC-T^sp^, teal: BMC-T^dp^, purple: signature enzyme, orange: regulator, red: aldehyde dehydrogenase (AldDh), green: alcohol dehydrogenase (AlcDh), magenta: phosphotransacetylase (PTAC), black: other conserved locus proteins, grey: genomic environment. CA: carbonic anhydrase

Bacterial microcompartments (BMCs) are protein-based organelles found in more than half of all bacterial phyla [1]. They consist of a shell, a membrane made of protein, that encapsulates a segment of a metabolic pathway (Fig. 1a). Sequestration of enzymes in BMCs has several prospective benefits such as separating sensitive enzymes from cytosolic conditions, preventing detrimental side reactions, protecting the cytosol from toxic intermediates, and improving reaction efficiency. The most extensively characterized BMC is the carboxysome that encapsulates carbonic anhydrase and RuBisCO to fix CO_2_ (Fig. 1a) [2]; carboxysomes are found in all cyanobacteria and some heterotrophic bacteria. A more diverse group of BMCs, termed metabolosomes, catabolize various substrates (such as choline, ethanolamine, 1,2-propanediol) via an aldehyde intermediate (Fig. 1a) [3, 4]. The enzyme(s) responsible for generating the aldehyde are termed signature enzyme(s). A recent bioinformatic survey uncovered many BMCs of unknown function [1], including some that lack the commonly found aldehyde dehydrogenase, suggesting that new paradigms for BMC-based reaction mechanisms await characterization.

The genes coding for BMCs are generally organized in an operon (BMC locus) that contains genes for structural proteins of the organelle as well as accessory factors like membrane transporters that bring substrate into the cell and regulatory proteins (Fig. 1b). In addition to a main locus, there are occasionally BMC-associated genes found in a distal, satellite location (Fig. 1b) [1, 4]. BMC loci can be detected in genomes by searching for the presence of the shell proteins that belong to pfam00936 and pfam03319. These proteins function exclusively in the context of BMCs and are sufficient to form the complete BMC shell. The pfam03319 protein, BMC-P, forms a pentamer in the shape of a truncated pyramid and is responsible for capping the polyhedral, often icosahedral shell (Fig. 1a, c) [5, 6]. The pfam00936 members are much more diverse; the most basic structure consists of six subunits of the BMC-H protein arranged in the shape of a regular hexagon; these form the bulk of the BMC shell facets (Fig. 1a, c) [7]. The hexamers have a diameter of about 65 Å and have a convex inward facing and an outward facing concave side (Fig. 1c). A circular permutation that involves two secondary structure elements that are displaced from the C- to the N-terminus is called BMC-H^p^; despite the displacement, the overall shape and size of the hexamer is preserved. Another shell protein variant is formed by a linear fusion of two copies of the pfam00936 domain and is known as BMC-T^s^; the fusion of two permuted pfam00936 domains is called BMC-T^sp^. Both types of BMC-T proteins form trimers that have a similar overall shape and edge lengths that match the hexamers (Fig. 1c). A more specialized form of BMC-T^sp^ forms trimers that dimerize across the concave face and is called BMC-T^dp^ (Fig. 1c) [8]; the dimerization forms internal chamber that is gated on both sides by highly conserved residues at the cyclic symmetry axis.

In a recent publication we described the use of a collection of protein profile HMMs to cluster BMC types. This has enabled identification of 68 BMC functional types or subtypes across 48 bacterial phyla [1]. While the profile HMMs are publicly available, here we make the analysis do-it-yourself by providing a web server that performs the analysis and formats the output with links to further, more detailed analysis of the protein components. Additionally, we provide a reference database for all the BMC types from our publication with linked detailed information and protein sequence download for further analysis by the user. While there is a database that collates the structures of BMC shell proteins available in the PDB (https://mcpdb.mbi.ucla.edu/) [9], there is no available interactive resource for BMC function prediction or a BMC type database. Determining the BMC type(s) is important to understand its role in an organism’s metabolism in its niche, from the human microbiome [10] to environmental detritus [1, 11].

## Construction and content

Protein sequence data were obtained by querying Uniprot (https://www.uniprot.org/) [12] for all BMC shell protein sequences (i.e. containing pfam00936 or pfam03319 domains). After extraction of the gene identifier for the BMC shell protein sequences, all proximal genes (± 12 genes) were downloaded as well. For each shell protein type (Fig. 1c, BMC-P, BMC-H, BMC-H^p^, BMC-T^s^, BMC-T^sp^, BMC-T^dp^), a phylogenetic tree was constructed and protein sequences from major branches were used to build profile HMMs named by shell protein type and a unique color name. Profile HMMs were also built for other gene products associated with BMCs. Additional details can be found in [1].

Each BMC locus was then scored against the HMM profile library to create a fingerprint that consists of the identity of best scoring protein HMMs, as well as matching their order on the chromosome. A simple cross-correlation was then calculated and used to cluster similar BMC loci. These BMC locus clusters were also compared to previously assigned BMC types [4] to ensure consistency and new BMC types named using the same general scheme as in [4].  After all BMC types were assigned, a set of BMC-type specific HMM profiles were calculated with each using sequences from only one BMC type. Those HMM profiles are then useful to identify a specific BMC-type. When almost all profile HMMs match to only one BMC type, a positive identification is highly likely.

The analysis of user sequences is performed by passing it as text to a python script in a cgi-bin directory of an Apache2 server running on a Red Hat Enterprise Linux virtual machine. The python script then performs a validity check on the input text and stores it as a file that is passed on to analysis with hmmsearch from the HMMer package [13]. The output file is then parsed by the python script and visualized as an HTML page.

In addition to a method to identify BMC types using protein HMM profiles, BMC Caller provides a complete database of more than 7000 BMC loci identified from the original Uniprot dataset described in [1] as pre-generated HTML pages with link for further analysis. They are sorted by BMC type and the type assignment has been manually verified.

## Utility and discussion

Due to the homology among BMC shell proteins, to-date automated annotations of sequence data typically assign proteins containing the pfam00936 or pfam03319 domains to the few extensively characterized BMC types (EUT, PDU and carboxysome). It is now clear that a function-based designation for shell proteins is misleading; especially the most common (and essential) BMC-H proteins often occur as multiple paralogs in a BMC locus and are found in different positions on a BMC-H phylogenetic tree [1, 14]. On this BMC-H tree the basal members are expected to form the bulk of the protein shell, while ones located on longer branches are likely to have a more specialized function [1, 14]. Our detailed sub-classification of the pfam00936 and pfam03319 proteins is also much more likely to detect connections between BMC types, for example the shell protein colors of the PDU1AB and GRM4 types are identical, indicating a common origin of those two types.

**Fig. 2:**
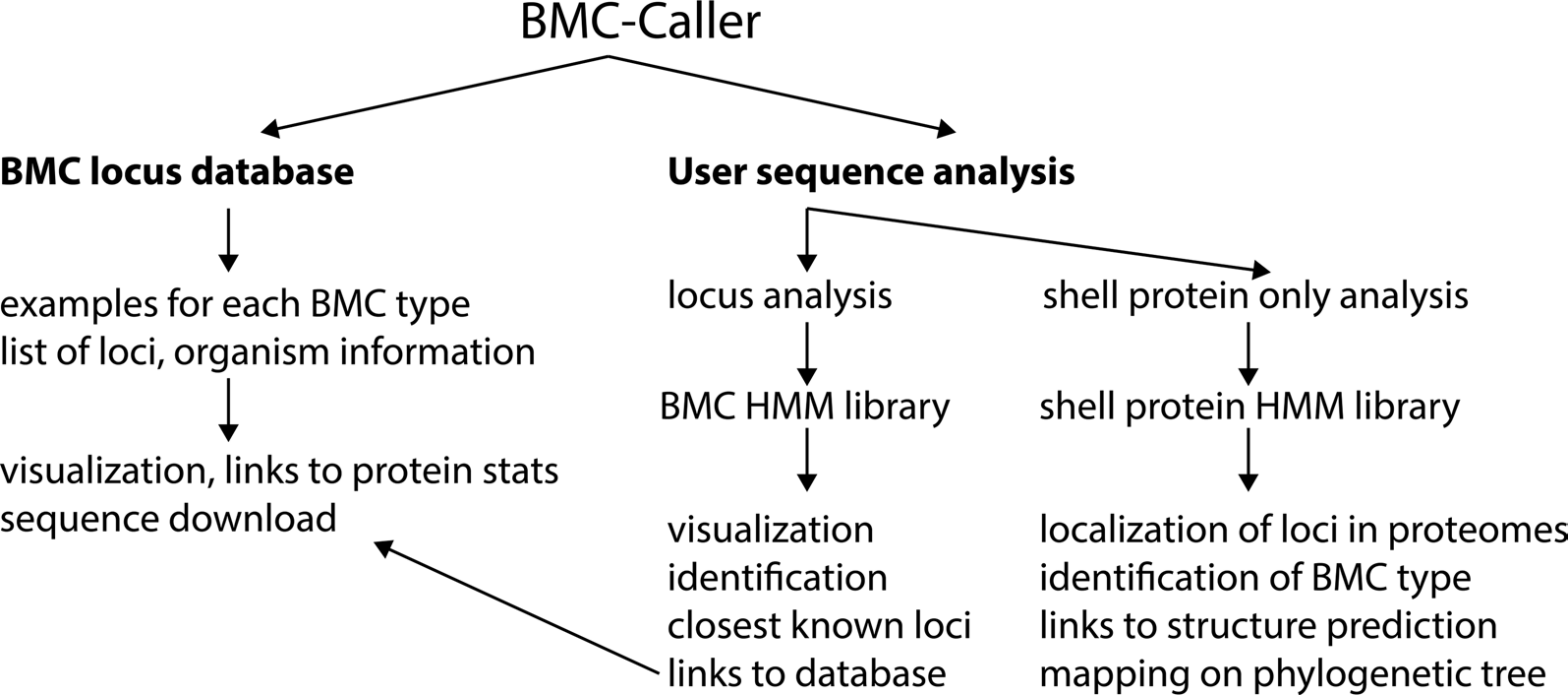
Overview of BMC Caller modes and result types

Our HMM protein profiles are able to distinguish 68 different BMC types or subtypes and will give the user information on homologs that share the same type. A short summary of BMCs is displayed on the landing page with links to the different modes of BMC Caller (Additional file 1: Fig. S1a). There is a database of the existing BMC types with information for all loci identified in [1, 14] as well as two modes that analyzes user sequences (Fig. 2).

### Sequence analysis modes

There are two modes for sequence analysis in BMC Caller, (1) analysis of all BMC protein components (includes the shell, the core enzymes and any other conserved loci-associated proteins) and (2) a BMC shell protein-only mode. Both modes accept multi-FASTA files as input and provide HTML output with links to further information for protein matches. The BMC locus type analysis mode contains an input field for protein sequences, a link for an example input, display options as well as a reset and submit button (Additional file 1: Fig. S1b). Display options include a choice of linear or cascading view, the former is preferable for viewing a large number of sequences and the latter is better suited for common lengths of BMC loci that consist of genes coding for about 20–30 proteins.

The output of the locus type analysis (Additional file 1: Fig. S1b) consists of:

- BMC type assignment based on the most common BMC type from the matches against the type-specific HMMs.

- A locus diagram display that lists all proteins with best HMM match and links to more details about the sequences constituting those HMMs. Details include the number of sequences in the HMM, their length and isoelectric point distribution, a sequence conservation logo as well as a link to download the sequences in FASTA format. The length of the bars in the locus diagram is scaled to protein sequence length and a color coding enables quick identification of different types of protein components. The protein identifier text with HMM assignments are colored green if they correspond to the most common BMC type and red otherwise.
- Closest related BMC types: this is based on comparison of both component inventory as well as the order of components to calculate a correlation score. The best five matches are shown as well as links to display their locus diagrams.
- A summary of shell proteins present with links to map them on the respective phylogenetic tree. This is useful to evaluate what shell proteins are most closely related. For the BMC-H tree a location of the protein close to the base of the tree frequently indicates that it is the major component of BMC shell facets [1, 14].

The locus analysis mode is best used in situations where the user knows the approximate extent of the BMC locus and provides only the sequences from within about 12 genes up- and downstream of the BMC shell proteins. For applications in which the user provides a whole proteome it is useful to first identify only the shell proteins which are diagnostic of BMC loci.

The BMC shell protein analysis mode uses only shell protein HMM profiles and can quickly identify them in whole proteomes (typically in less than a minute). The shell protein HMMs are also BMC type specific so they are likely to also identify the BMC type. The output provides all the shell proteins along with their FASTA identifiers in a list mode with links to their HMMs analogous to the locus mode output (Additional file 1: Fig. S1c). There are a variety of protein structures available in the Protein Data Bank for each shell protein type so homology models are quite reliable; for this purpose we have added a direct link to generate a homology model with SWISS-MODEL [15]. Shell protein models provide information about the potential permeability of the shell, which can provide clues to the size and charge of molecules that traverse the shell, for example as products or substrates of the encapsulated reactions (Fig. 1a). The analysis also shows a summary of the shell proteins by type with link to visualize them on the respective phylogenetic trees.

If further BMC type analysis is needed the user can use the genomic regions around those identified shell proteins for the BMC locus analysis, provided that the protein sequences are ordered the same as on the genome.

### Database mode

The locus database mode of BMC Caller gives an overview of the different BMC types and subtypes as presented in [1]. Each type/subtype has an example locus diagram that has further links to all loci of that type as well as which proteins are commonly found in those types. Direct links to the sequence files are available for all proteins to enable further analysis like sequence alignment or structure prediction.

### Case study of an unknown BMC type

Our comprehensive HMM profile database of all currently known BMC types is also useful in identifying new BMC types. Here we demonstrate this by scoring a recent collection of metagenome assembled genomes (MAGs) [16] with our profile HMMs. Many genomes with positive hits for BMC shell proteins show a consistent pattern of matching against a single BMC type, indicating the presence of a BMC locus of that type. However, some of the ambiguous assignments are potentially new BMC types or subtypes. An example of this is the metagenome assembled genome from a Firmicute found in a switchgrass degrading bioreactor metagenome. The analysis of the proteome of that MAG containing 2772 sequences shows mixed BMC type assignments of the shell proteins, containing representatives found in SPU5, ACI, SPU4, SPU1, EUT2D and SPU6 types (Additional file 1: Fig. S2a); this further highlights the importance of using a function agnostic shell protein naming scheme [1, 14]. Extracting just the region around the BMC shell proteins still results in a set of mixed type assignments and there is no single best matching BMC type (Additional file 1: Fig. S2b). However, two of the matches are labeled as signature enzymes (purple) and they belong to the SPU (sugar phosphate utilization) type, indicating that this might be its primary function. A SWISS-MODEL [15] structure prediction started for the H_azure and H_fuchsia type shell proteins via the provided links maps the protein sequences onto hexameric homology models. Visualization of the residues surrounding the pore with PyMOL (https://pymol.org) (Additional file 1: Fig. S2c) shows six lysines lining the pore of the H_azure hexamer, and six tyrosines surrounding the pore in the H_fuchsia hexamer. The latter is a less common BMC-H protein and has an N-terminal extension, so is likely responsible for a more specialized function. The H_azure BMC-H is expected to be the major shell protein based on its location closer to the base of the BMC-H tree and a pore lined with lysine residues is consistent with the hypothesis that the substrate, putatively a sugar phosphate, is negatively charged.

It is likely that this is a new SPU subtype that could be integrated into the database by generating profile HMMs, therefore simplifying future identification as a SPU subtype. For this it is necessary to find more homologues, ideally 3 or more to generate suitable alignments for HMM generation.

We are planning to update BMC Caller with new BMC types from our own investigations as well as user submitted new BMC types to serve the research community and ensure consistent naming of BMC types as the database of known functional types expands.

## Conclusions

The goal of BMC Caller is to make BMC type prediction and analysis available to the diverse scientific community. The BMC Caller is available at https://bmc-caller.prl.msu.edu. We expect that with the exponential increase in genomic sequence data there are more BMC types yet to be discovered and a reference to the existing BMC types will greatly facilitate this.

## Supplementary Information

**Additional file 1: Fig. S1**. Overview of interface and result pages. **a** Landing page with brief introduction to BMC structure and function. **b** Submission form for locus type analysis and example result page of locus analysis. Bar length is scaled to gene length. **c** Example result page of shell protein analysis. **Figure S2**. Example output for a novel type of BMC. **a** BMC shell protein analysis of the whole proteome of this metagenome assembled genome (MAG) with id 3300010269_9. **b** BMC locus analysis of the region around the shell proteins from Ga0134102_1000278_11 to Ga0134102_1000278_29. **c** Close-up view of pores in the homology model structures predicted with SWISS-MODEL for the H_azure (based on pdb ID 3MPY) and H_fuchsia (based on pdb ID 4AXJ) The residues converging at the pores of the hexamers are shown in sticks.

## Data Availability

Project name: BMC Caller. Project home page: https://bmc-caller.prl.msu.edu. Operating systems: Platform independent. Programming language: Python. Other requirements: Web browser. License: None. Any restrictions to use by non-academics: None.

## Abbreviations

BMC: Bacterial microcompartment
AldDh: Aldehyde Dehydrogenase
AlcDh: Alcohol Dehydrogenase
PTAC: Phosphotransacetylase
MAG: Metagenome assembled genome
