# Supplementary material for: BMC Caller: a webtool to identify and analyze bacterial microcompartment types in sequence data
Source: Biol Direct. 2022 Apr 28;17:9. doi: 10.1186/s13062-022-00323-z (PMC9052549; doi:10.1186/s13062-022-00323-z)
Supplement: Supplementary file 1 — Additional file 1: Fig. S1. Overview of interface and result pages. a Landing page with brief introduction to BMC structure and function. b Submission form for locus type analysis and example result page of locus analysis. Bar length is scaled to gene length. c Example result page of shell protein analysis. Figure S2. Example output for a novel type of BMC. a BMC shell protein analysis of the whole proteome of this metagenome assembled genome (MAG) with id 3300010269_9. b BMC locus analysis of the region around the shell proteins from Ga0134102_1000278_11 to Ga0134102_1000278_29. c Close-up view of pores in the homology model structures predicted with SWISS-MODEL for the H_azure (based on pdb ID 3MPY) and H_fuchsia (based on pdb ID 4AXJ) The residues converging at the pores of the hexamers are shown in sticks. [file 13062_2022_323_MOESM1_ESM.pdf]

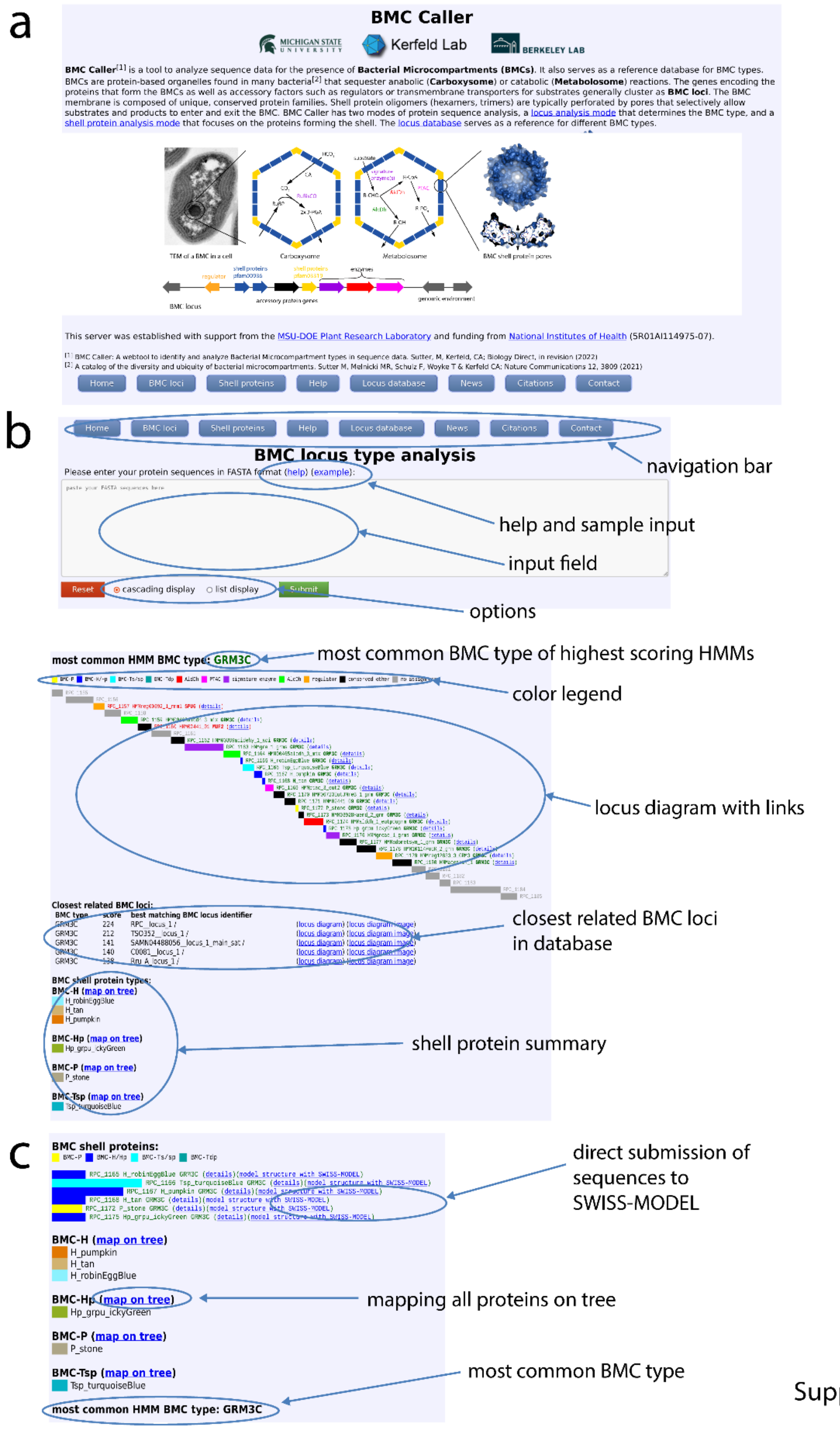

Supplementary Figure S1

Supplementary Figure S1. Overview of interface and result pages. a) Landing page with brief introduction to BMC structure and function. b) Submission form for locus type analysis and example result page of locus analysis. Bar length is scaled to gene length. c) Example result page of shell protein analysis.

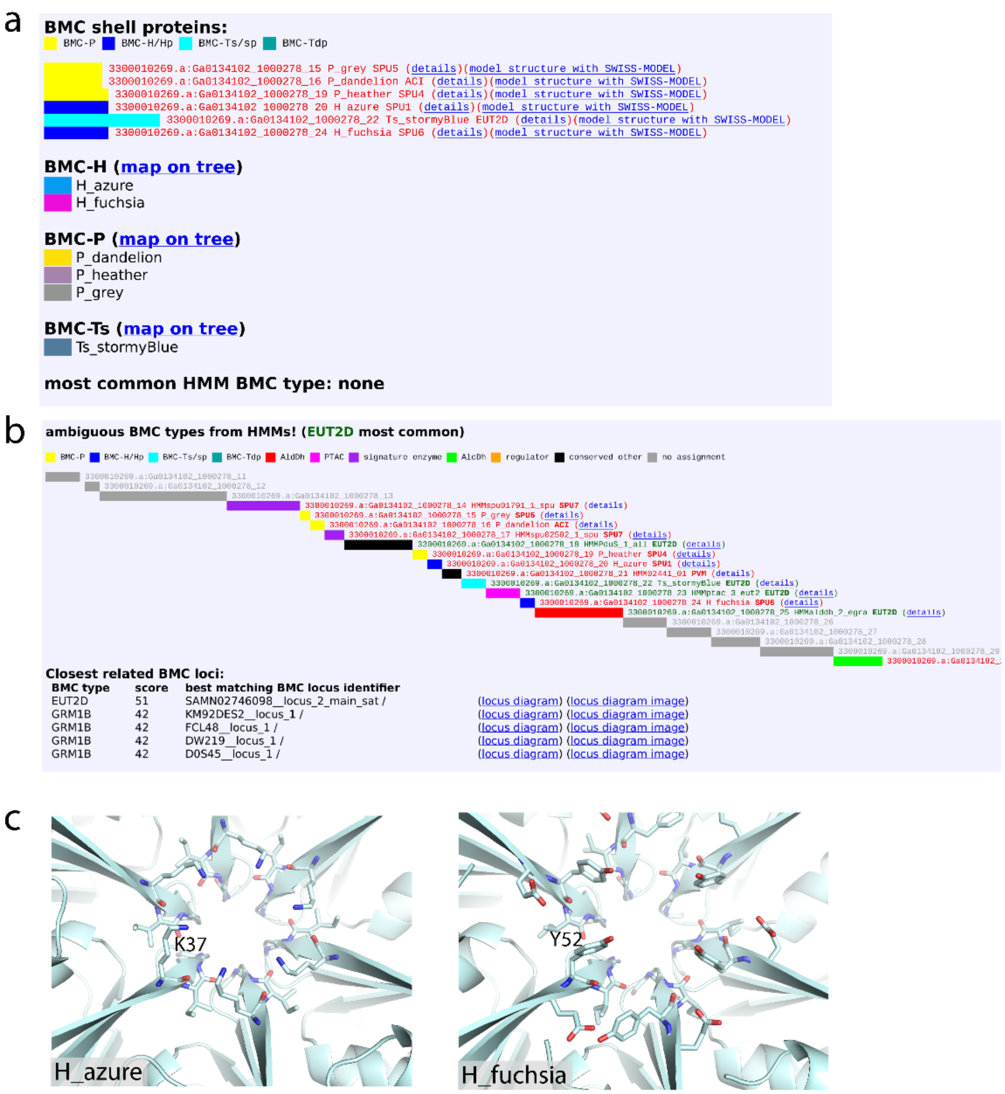

Supplementary Figure S2

Supplementary Figure S2. Example output for a novel type of BMC. a) BMC shell protein analysis of the whole proteome of this metagenome assembled genome (MAG) with id 3300010269_9. b) BMC locus analysis of the region around the shell proteins from Ga0134102_1000278_11 to Ga0134102_1000278_29. c) Close-up view of pores in the homology model structures predicted with SWISS-MODEL for the H_azure (based on pdb ID 3MPY) and H_fuchsia (based on pdb ID 4AXJ) The residues converging at the pores of the hexamers are shown in sticks.
